# Integrating Assisted Reproductive Technologies, Biobanking, and Genomic Rescue for Endangered Wildlife: A Vertebrate-Focused Review

**DOI:** 10.3390/ijms272110050

**Published:** 2026-11-09

**Authors:** Imran Khan, Atif Ali Khan Khalil, Ruttachuk Rungsiwiwut, Sirisuda Subboriboon, Kaywalee Chatdarong, Ampika Thongphakdee, Rangsun Parnpai

**Affiliations:** 1 Embryo Technology and Stem Cell Research Center, School of Biotechnology, Suranaree University of Technology, Nakhon Ratchasima 30000, Thailand; imrangnu@gmail.com (I.K.); jasmine_sirisuda@kkumail.com (S.S.); 2 Department of Biotechnology, Yeungnam University, Gyeongsan 38541, Gyeongbuk, Republic of Korea; atif.ali@yu.ac.kr; 3 Department of Anatomy, Faculty of Medicine, Srinakharinwirot University, Bangkok 10110, Thailand; ruttachuk@g.swu.ac.th; 4 Department of Obstetrics, Gynaecology and Reproduction, Chulalongkorn University, Bangkok 10330, Thailand; kaywalee.c@chula.ac.th; 5 Animal Conservation and Research Institute, The Zoological Park Organization of Thailand, Bangkok 10300, Thailand

**Keywords:** wildlife conservation, artificial insemination, somatic cell nuclear transfer, induced pluripotent stem cells, in vitro gametogenesis, conservation genomics

## Abstract

Assisted reproductive technologies (ART) provide opportunities for conservation of endangered wildlife, but their use is still limited by species-specific reproductive physiology, variable gamete competence, cryoinjury, and lack of validation of new approaches. This narrative review covers ART, biobanking, somatic cell nuclear transfer (SCNT), induced pluripotent stem cells (iPSCs), in vitro gametogenesis (IVG) and conservation genomics in vertebrates. Key molecular barriers include oxidative and mitochondrial damage, aberrant embryonic genome activation, incomplete epigenetic reprogramming, nuclear-mitochondrial incompatibility and defective germ-cell differentiation. Technological readiness is used to evaluate the evidence and separate demonstrated reproductive outcomes from experimental proof of concept, and indirect findings from domestic or laboratory models. Semen cryopreservation and artificial insemination are established in selected species, while embryo-based interventions remain species-dependent and stem-cell-derived reproductive approaches require functional validation. We propose an integrated decision framework linking reproductive diagnosis, biobanking, genomic donor selection, technological readiness, animal welfare, and resource allocation with reproductive and population-level outcomes. Importantly, laboratory milestones alone cannot demonstrate conservation success without sustained genetic contribution across multiple subsequent generations. Successful translation depends on species-specific protocols, standardized endpoints, and evidence of genetically valuable offspring survival, reproduction, and contribution to long-term population viability.

## Simple Summary

Habitat protection and natural breeding alone will not be enough to recover many endangered species that exist in small, isolated populations. Assisted reproductive technologies have the capacity to preserve and transfer valuable genetic material across individuals, places and generations. These include artificial insemination, cryopreservation of gametes and tissues, production and transfer of embryos, cloning and stem-cell-based approaches. Biobanks are collections of biological material for future use, with genomic information supporting the selection of donors and genetic rescue. But offspring production does not guarantee population recovery. Offspring should be healthy and fertile, genetically valuable, and able to produce viable populations. This review highlights the applications, limitations and technology readiness of these technologies within the major vertebrate groups. Animal welfare, governance, resource allocation and long-term monitoring are also considered. Reproductive technology should be combined with conservation of habitat, reduction of threats, managed breeding and ecological restoration.

## 1. Introduction

Many species now survive as small, isolated populations affected by interacting demographic pressures, including demographic stochasticity, skewed sex ratios [1], reproductive senescence [2] and loss of genetic diversity [3]. Protection of habitat, reduction of threats, translocation and managed breeding are still the basis of conservation, but these do not always solve the problem of reproduction. Potential mates may be housed at different institutions or geographically separated. Natural mating may also be hazardous or repeatedly unsuccessful, and genetically valuable animals may die before reproducing. In these cases, reproductive biotechnology can turn stored or otherwise unavailable biological material into a future opportunity for breeding [4]. Its value is not just in producing individual young. It can facilitate genetic exchange without transporting whole animals, extend reproductive opportunities across generations, and enable the recovery of underrepresented founder lineages through assisted reproduction and the use of preserved biological materials [5,6].

Assisted reproductive technologies (ART) include reproductive monitoring, endocrine manipulation, semen collection and storage, artificial insemination (AI), oocyte retrieval, in vitro maturation (IVM), in vitro fertilization (IVF), intracytoplasmic sperm injection (ICSI), embryo culture, embryo transfer (ET), gonadal-tissue preservation and related micromanipulation techniques. The technical difficulty and biological burden of these approaches range widely. IVF and subsequent ET require the coordination of gamete quality, embryo development, uterine physiology, and recipient management. AI allows the genetic contribution of a male to be introduced with relatively limited intervention. More experimental approaches may present future opportunities for recovering genetic variation from viable cells in cases where usable gametes are unavailable, including somatic cell nuclear transfer (SCNT) [5], induced pluripotent stem cells (iPSCs) [4], primordial germ-cell (PGC) technologies [7] and in vitro gametogenesis (IVG) [8]. Most are still at experimental or proof-of-concept stages, however.

Incomplete epigenetic reprogramming is an important molecular barrier to the development of SCNT embryos. Analysis of deoxyribonucleic acid (DNA) methylation in primate SCNT embryos revealed widespread methylation abnormalities and loss of imprinting in maternally imprinted genes [9]. Interspecies SCNT can further restrict embryonic development because of incompatibility between the donor nucleus, recipient-oocyte mitochondria, and cytoplasmic reprogramming factors [10]. Primordial germ-cell-like cells have been generated from induced pluripotent stem cells of the northern white rhinoceros, but their capacity to complete meiosis and produce functional gametes has not been demonstrated [7]. These results indicate that molecular evaluation must be used in combination with morphological evaluation in selecting donor cells, gametes, embryos and surrogate species for conservation programs.

**Figure 1:**
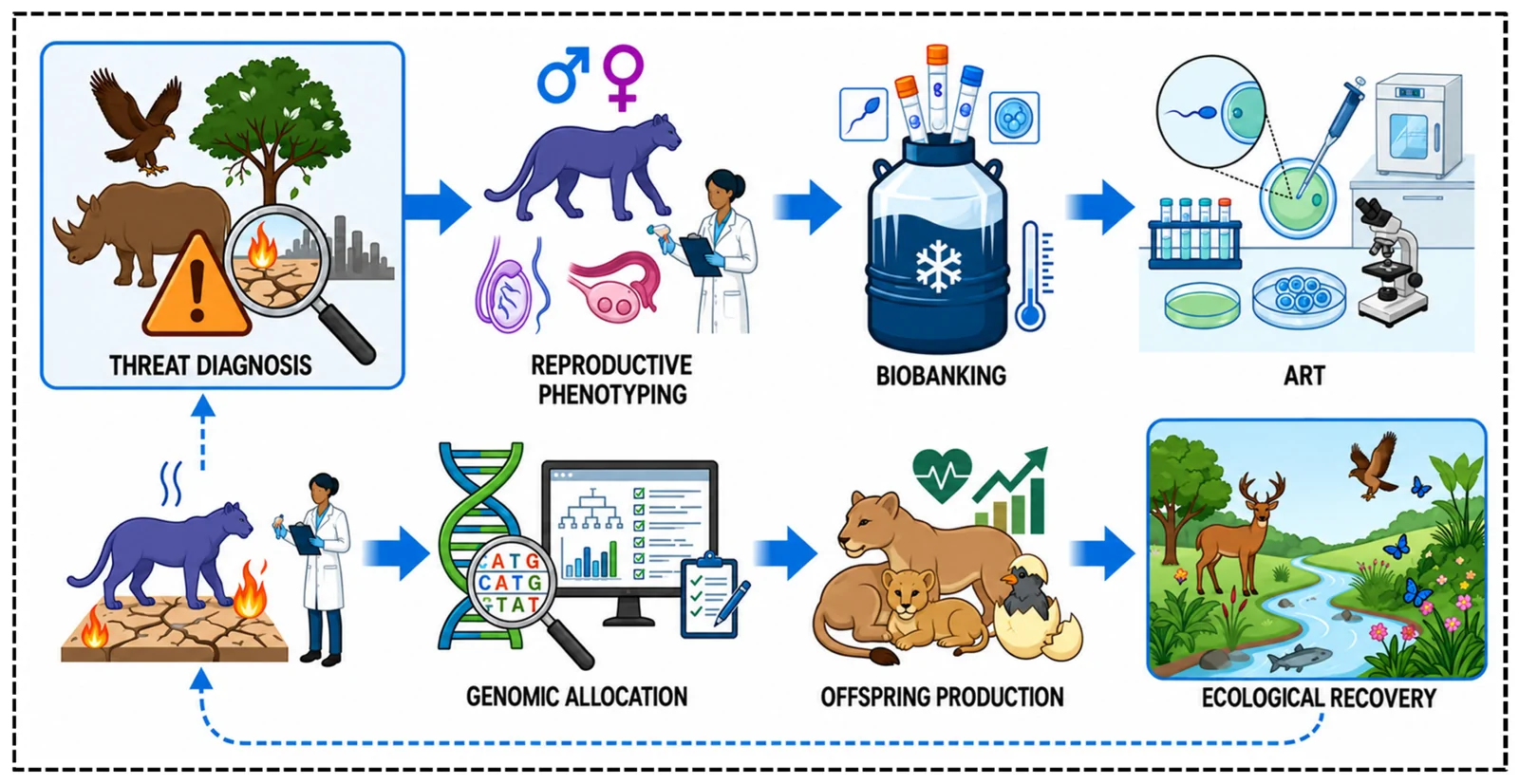
Integrated framework linking reproductive biotechnology with endangered-wildlife recovery. The conceptual framework depicts the links between threat diagnosis, reproductive phenotyping, biobanking, assisted reproductive technologies (ART), genomic allocation, offspring production and ecological recovery. Solid arrows indicate the proposed flow between linked conservation activities. Dashed arrows indicate adaptive feedback from long-term monitoring and management to previous conservation assessments. The illustrated components are representative of the complementary functions within an integrated conservation program and not a sequential or experimentally validated pathway. Long-term reproductive, genetic, and population monitoring informs adjustments to future conservation strategies.

Successful application of a reproductive technique in the laboratory does not necessarily prove its effectiveness for conservation. Even if sperm remains motile, an embryo undergoes cleavage, or a live animal is born, the risk of extinction may not be reduced unless the offspring demonstrates normal development, health, fertility, species-appropriate behavior, reproductive contribution, and, where possible, favorable multigenerational outcomes. Conversely, even a method with modest technical efficiency may be valuable when it enables the recovery of an underrepresented founder lineage or introduces genetic contributions from previously underrepresented breeders [6]. Success should be measured by its contribution to population viability and not just laboratory milestones [11]. Figure 1 presents a general overview of how reproductive assessment, biobanking, ART, genomic allocation and long-term monitoring are integrated in a conservation program.

The field is moving from isolated rescue attempts to coordinated, multistage conservation programs. For example, the work on the northern white rhinoceros includes repeated ovum pick-up, ICSI, embryo cryopreservation, preparation of surrogate females [12] and stem-cell research [7]. Cloning of Przewalski’s horses and black-footed ferrets has shown that fibroblast lines preserved for decades can recover underrepresented nuclear genetic variation. But genetic rescue can only be demonstrated when that variation is inherited by reproductively successful descendants and contributes to future generations. The development of avian primordial germ cell culture and cryobanking is expanding the reproductive toolkit for bird conservation [13]. Germ cell cryobanking and transplantation offer complementary strategies for preserving genome-wide diversity and generating donor-derived gametes in threatened fishes [14]. Production of live offspring from cryopreserved sperm of the tiger salamander also demonstrated the potential to apply sperm biobanking to amphibian conservation [15].

Assisted reproductive technologies (ART), biobanking, cloning and stem-cell-based approaches in endangered-species conservation have been reviewed previously [16,17]. The studies provide important assessments of individual technologies, their biological limitations and possible conservation applications. Yet a major challenge is to embed this evidence into a decision-oriented framework that explicitly links reproductive barriers to intervention choice, species-specific technological preparedness, animal welfare, genetic management and quantifiable conservation results. To address this gap, the present review proposes an integrated framework consisting of six interconnected components: (1) reproductive and genomic assessment to identify biological limitations and genetically valuable individuals; (2) intervention selection based on diagnosed reproductive barrier, technical feasibility, and available biological resources; (3) evaluation of technological readiness using species-specific functional evidence; (4) coordinated biobanking and genomic donor allocation to preserve and utilize underrepresented genetic variation; (5) assessment of animal welfare, recipient compatibility, resource requirements, and implementation feasibility; and (6) monitoring of offspring health, fertility, genetic contribution, and long-term population recovery. These components are intended to guide conservation decisions, not to represent a universally validated intervention algorithm. Their primary contribution is to link reproductive and molecular evidence to successive conservation decisions and downstream consequences, while distinguishing laboratory feasibility from documented genetic, demographic, and ecological benefits.

Conceptually, genetic rescue is defined as an increase in population fitness as a consequence of the addition of genetic variation. Conversely, in this review we adopt the term genomic rescue as an operational definition to describe genomics-informed strategies to maintain, restore, or redistribute genetic variation without necessarily implying demonstrated improvements in population fitness. Genomic allocation is described, in the scope of the proposed framework, as the selection of genetically important donors, biological materials and breeding combinations using genomic information such as relatedness, founder representation and genetic diversity. These distinctions can be illustrated using examples from vertebrate conservation. Genetic supplementation in Florida panther resulted in long-term genetic and demographic benefits over several generations, providing direct evidence for genetic rescue [11]. Cloning of Przewalski’s horses from historically cryopreserved somatic cells has produced healthy offspring with an underrepresented founder genome, but reproductive and population-level contributions are still to be determined [5]. Genetic sequencing, demographic reconstruction and environmental analysis were used to inform assessment of potential donor populations for genetic rescue in montane red foxes. This was an example of genomics-informed intervention planning, not demonstrated rescue outcomes [18]. Hence, genomic allocation supports conservation decision-making, genomic rescue describes genomics-informed conservation strategies and genetic rescue requires demonstrated beneficial population outcomes.

## 2. Literature Search and Review Methodology

This narrative review was built on studies identified via PubMed and Google Scholar, with a literature-search cutoff of 3 October 2026. The search focused on assisted reproductive technologies, cryopreservation, biobanking, conservation genomics and genetic rescue in endangered wildlife. The search concepts included combinations of terms such as endangered species, wildlife conservation, assisted reproductive technologies, artificial insemination, sperm cryopreservation, oocyte maturation, in vitro fertilization, intracytoplasmic sperm injection, embryo transfer, somatic cell nuclear transfer, induced pluripotent stem cells, in vitro gametogenesis, germ-cell transplantation, biobanking and genetic rescue. The review focused on major vertebrate groups, including mammals, birds, fishes, amphibians, and reptiles, while drawing on relevant domestic and laboratory models where these provided mechanistic or experimental evidence applicable to wildlife conservation.

Studies were selected for inclusion based on relevance to reproductive feasibility, species-specific biological constraints, technological readiness and conservation outcomes. Primary research reporting experimental procedures, molecular or cellular findings, gamete and embryo development, pregnancy, offspring production, or genetic and population-level outcomes were emphasized. Reviews that were relevant were also considered for the wider scientific context. Studies on endangered wildlife received special consideration, while evidence from domestic or laboratory species was included when it provided mechanistic insight or experimental approaches relevant to wildlife reproduction. Publications that lacked enough relevance to reproductive biotechnology or conservation, or lacked enough scientific information to sustain the discussed claims, were not emphasized in the synthesis.

The literature was critically interpreted based on the species studied, the reproductive intervention studied and the highest functional outcome demonstrated. Direct evidence from target wildlife species and studies reporting functional reproductive outcomes were afforded greater interpretative weight, whereas molecular findings, experimental proof-of-concept studies, and evidence from domestic or laboratory models were distinguished from validated conservation applications. This review is based on a narrative synthesis and not a systematic review or meta-analysis, and thus no formal quantitative study-selection or risk-of-bias assessment is claimed. The synthesis that emerges combines reproductive, molecular and conservation evidence into a decision-oriented framework, while highlighting species-specific limitations and priorities for further validation.

## 3. Reproductive Diagnosis and Technology Selection

Conservation programs should be based on reproductive diagnosis and not on a preferred technology. The first question is whether breeding failure is due to mating incompatibility, social environment, nutrition, disease, endocrine dysfunction, gamete defects, age or simply bad timing. Longitudinal measurements of reproductive hormones can be useful in characterizing reproductive activity, but for each species, the sampling matrix and analytical assay need to be validated. For example, enzyme immunoassays for fecal androgen, estrogen and progestogen metabolites were biologically validated for non-invasive reproductive monitoring in Temminck’s pangolin [19]. Species-specific interpretation is essential [20] since seasonality, induced ovulation, delayed implantation, embryonic diapause and spontaneous pseudopregnancy can make domestic-animal assumptions invalid.

The male reproductive assessment should include sperm concentration, motility, morphology, plasma-membrane and acrosomal integrity, mitochondrial activity and DNA fragmentation. Validated functional assays for these endpoints have been developed in the endangered golden-headed lion tamarin and have shown applicability beyond routine semen evaluation [21]. In wildlife, the act of collecting can change the quality of the sample [22]. Electroejaculation, urethral catheterization after pharmacological stimulation, manual collection, post-coital recovery and epididymal retrieval all impose different welfare, logistical and physiological constraints [23]. Opportunistic samples taken after death can still be useful, but the amount of usable sperm and oocytes obtained is heavily influenced by post-mortem interval [23], tissue temperature and the conditions in which they are transported. The assessment of females is often the bottleneck, because ovarian access, anesthesia and synchronization are more invasive and less standardized [24].

**Figure 2:**
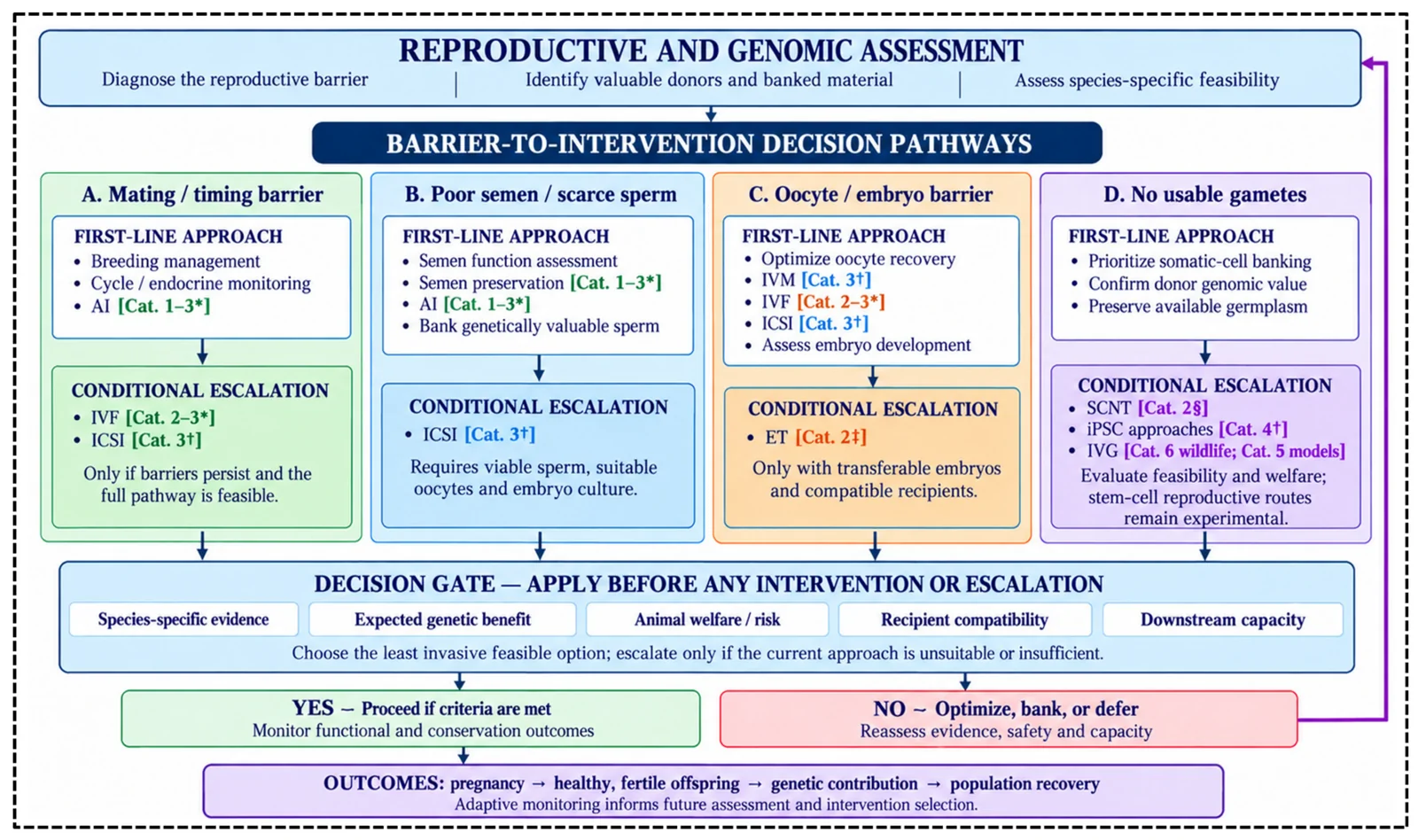
Decision framework for selecting reproductive interventions in endangered-wildlife conservation. Four pathways connect diagnosed reproductive barriers with first-line approaches and conditional escalation options. The annotations refer to the evidence-readiness categories defined in Section 3. Asterisks indicate species-dependent ranges: artificial insemination (AI) and semen preservation span Categories 1–3, from routine use in selected conservation programs to gamete-level proof of concept, while in vitro fertilization (IVF) spans Categories 2–3, from live-offspring production to embryo-level proof of concept. Daggers (†) indicate rhinoceros evidence supporting Category 3 for in vitro maturation (IVM) and intracytoplasmic sperm injection (ICSI), and Category 4 for induced pluripotent stem cell (iPSC) approaches. The double dagger (‡) identifies Category 2 evidence from live cheetah offspring following embryo transfer (ET), and the section symbol (§) identifies Category 2 evidence from cloned Przewalski’s horses produced by somatic cell nuclear transfer (SCNT). These examples do not establish equivalent readiness in other target species. In vitro gametogenesis (IVG) remains a proposed wildlife application (Category 6), with indirect evidence from laboratory models (Category 5). Before intervention or escalation, the decision gate considers species-specific evidence, expected genetic benefit, animal welfare, recipient compatibility, and downstream capacity.

The technology-readiness profile should contain a series of related requirements such as reliable reproductive-cycle staging, safe anesthesia, access to gametes, validated extenders and cryoprotectants, sufficient culture conditions, benchmarks for embryo development [12], recipient synchronization, pregnancy diagnosis, and neonatal management. If a failure occurs at any point, the intervention as a whole may become ineffective. Figure 2 illustrates the proposed decision framework for selecting reproductive interventions based on reproductive status, genetic value, technical feasibility, and animal welfare. Natural and assisted breeding should be considered complementary approaches, with assisted reproductive technologies providing additional opportunities for genetically valuable individuals [5] whilst improvements in conventional breeding management may benefit the wider population. Here, technological readiness is categorized based on available evidence: routine use in conservation; evidence of live-offspring production in the target or closely related species; proof of concept at the gamete or embryo level; proof of concept at the cellular or molecular scale; indirect evidence from domestic or laboratory models; and theoretical or future applications. Results from model species cannot be taken as proof of technological readiness in endangered wildlife without proper species-specific validation.

To differentiate between demonstrated reproductive outcomes, preliminary findings, and proposed applications, this review defines six evidence-readiness categories: (1) Category 1: Routine use in conservation, indicating repeated application within an established conservation program using species-specific protocols with documented functional reproductive outcomes; (2) Category 2: Live-offspring production in the target or closely related species, indicating that the specified intervention has produced live offspring, with the species explicitly identified, without necessarily establishing routine use, offspring fertility, or sustained genetic contribution; (3) Category 3: Gamete- or embryo-level proof of concept, indicating demonstrated gamete viability or function, fertilization, or embryo development without documented live-offspring production from the specified procedure in the species assessed; (4) Category 4: Cellular or molecular proof of concept, indicating demonstrated cellular or molecular outcomes, such as cell reprogramming, pluripotent-cell derivation, or early germ-cell differentiation, without demonstrated functional reproductive competence; (5) Category 5: Indirect evidence from domestic or laboratory models, indicating experimental findings that inform a potential wildlife application but do not validate that application in the target species; and (6) Category 6: Theoretical or proposed application, indicating a conservation application without direct experimental validation in the intended species and context. These categories are evidence for a given species and application, not a universal sequence of technological maturity. It is therefore necessary to state explicitly the species providing the evidence and the precise endpoint achieved. Findings from domestic or laboratory models provide indirect evidence for unvalidated wildlife applications, and category assignments do not independently establish feasibility of implementation, acceptability to animal welfare, or conservation benefit at the population level.

The proposed decision framework relates the diagnosed reproductive barrier to the least invasive intervention with sufficient species-specific evidence and realistic prospects of reproductive success. Where breeding failure is largely due to mating incompatibility or incorrect timing of reproductive activity, reproductive management, endocrine monitoring and validated artificial insemination should be considered prior to more invasive procedures. If the availability of sperm or its fertilizing capacity is limited, semen assessment and preservation should be the priority, and intracytoplasmic sperm injection should be considered only if compatible oocytes, embryo culture conditions and suitable recipients are available. Where oocyte competence, fertilization or embryonic development is the primary hurdle, embryo transfer should be preceded by targeted optimization of gamete and embryo procedures. Banking of somatic cells may preserve future reproductive options in genetically valuable individuals with no usable gametes. Somatic cell nuclear transfer and stem-cell-based reproductive approaches require separate evaluation of experimental readiness and welfare implications. At each decision point, the expected genetic contribution and reproductive benefit must outweigh the procedural burden, technological uncertainty and resource demands. Escalation should be based on the previous approach being inappropriate or inadequate and the subsequent intervention having sufficient biological evidence and downstream capacity. If these conditions are not satisfied, further validation, preservation of available biological materials, or deferral of the intervention are preferable to proceeding solely because a more advanced technology is available.

Bayesian [25] and value-of-information [26] reasoning can also assist in intervention design. When animals are scarce, the goal is not to maximize the number of procedures but to reduce the uncertainty that most strongly impacts a management decision. If the non-invasive endocrine series can provide resolution of cycle length [27] and ovulation timing, it may be more valuable than an immediate invasive collection. In contrast, the fact that post-mortem gonadal recovery is a non-repeating opportunity may justify rapid action [28]. Anesthesia, catheter geometry, media osmolality and sample handling should be determined with pilot work in a related species [29] before the enrollment of target animals. The resulting protocol should have success criteria at each stage and establish alternative uses for samples that fail the primary pathway, such as conversion of poor-quality tissue into fibroblast cultures [30] or genomic DNA.

Reproductive data must be integrated into population management software, not just kept as clinical records. The genetic contribution expected from each candidate is influenced by fertility probability, age decline, past contraception [31], uterine pathology and semen quality. By incorporating these variables with mean kinship [32], we can avoid over-investing in animals that are genetically common or biologically unlikely to reproduce. It also tells us when an underrepresented but sub-fertile individual merits disproportionate effort [6]. This type of triage is ethically preferable because it aligns procedural burden with conservation value and avoids the performance of technically impressive interventions that do not address the limiting factor of the population.

## 4. Semen Technologies and Artificial Insemination

The most well-documented areas of wildlife ART are semen collection, cryopreservation and artificial insemination, but routine use is restricted to species for which collection, storage and insemination protocols have been validated. Cryotolerance, however, varies dramatically among species and individuals [33]. Membrane lipid composition, osmotic sensitivity, seminal plasma, sperm morphology and antioxidant capacity influence how well sperm retain their fertilizing function during cooling and freezing. Extenders developed for cattle or dogs may provide a starting point, but conservation protocols require factorial testing of buffer, energy substrate, protein source, penetrating cryoprotectant, cooling rate and thawing regime [34].

Molecular and functional markers may offer additional information on post-thaw sperm quality that complements conventional motility assessment. In a domestic ram model of semen cryopreservation, supplementation with the mitochondria-targeted antioxidant MitoQ improved sperm motility, mitochondrial membrane potential, membrane functionality, acrosomal integrity and reproductive performance, while reducing oxidative stress, lipid peroxidation, DNA fragmentation and apoptosis-like changes [35]. These results suggest the possible advantages of targeting mitochondrial oxidative damage in ram sperm, but do not prove that this treatment works in endangered wildlife. Conversely, direct studies in endangered Eld’s deer showed cryopreservation-related changes in sperm protein profiles and decreased post-thaw motility, viability and membrane and acrosomal integrity [36]. Proteins involved in cryostress responses were also identified in the seminal plasma of Eld’s deer by proteomic analysis, offering some insights into the molecular alterations during semen preservation [37]. While these studies provide species-specific molecular evidence, the identified proteins remain candidate biomarkers rather than validated predictors of fertility. Therefore, molecular and functional assessments should be added to conventional semen evaluation. Antioxidant treatments and candidate biomarkers need to be validated in a species-specific way against fertilization and reproductive outcomes before being used in routine conservation.

In several threatened mammals and birds, AI has produced offspring, especially when ovulation can be predicted, and semen can be placed near the fertilization site [6]. The route vaginal, transcervical, intrauterine or laparoscopic should minimize restraint while delivering an adequate sperm dose. Semen cryopreservation can preserve reproductive material for future genetic exchange, although its application requires species-specific validation of post-thaw fertilizing capacity. Post-thaw motility alone is insufficient to establish reproductive competence. Functional assays and, where ethical justifications allow, controlled insemination trials are needed. Table 1 summarizes the principal sperm-based interventions and their reported conservation applications.

Scalability is the primary strategic advantage of semen banking. One male can contribute to several females and future cohorts, decreasing the need to transport territorial, dangerous or disease-sensitive animals. Yet paternal-only banking may not preserve mitochondrial genomes and may need repeated backcrossing to restore a lineage. Thus, programs should combine semen with oocytes, embryos, gonadal tissue and somatic cells whenever possible. Storage must be genomically informed to target samples that do not simply replicate common lineages [38].

Cryopreservation experiments should separate sources of variation that are often confounded in wildlife research. The male identity, season, collection method, initial quality, extender and freezing curve should be recorded separately. The split-ejaculate designs are particularly powerful because they compare treatments within the same donor, conserving samples and improving inference [39]. Computer-assisted sperm analysis can enhance objectivity, but algorithms trained on domestic species may misclassify unusual sperm head or flagellar patterns. Manual validation and species-appropriate chamber depth are still necessary. Molecular end points, including DNA oxidation, chromatin integrity and capacitation competence, can reveal damage invisible to routine motility measures [40]. Proteomic evaluation also identified candidate biomarkers related to sperm traits and fertility in three threatened wild felids [41]. These species-specific findings provide direct evidence of molecular biomarkers relevant to endangered species reproduction. The antioxidant responses reported in ram spermatozoa [35] are indirect evidence and need to be validated in threatened species.

The genetic consequences of AI depend on the insemination policy. Repeated use of a high-performing male can lead to faster loss of diversity even if conception rates are improved. Programs should limit lifetime offspring, allocate doses according to mean kinship [32] and preserve semen before a male becomes overrepresented. Sampling of frozen semen across age classes and locations should also be done because adaptive and rare alleles may not coincide with conventional measures of reproductive quality. A useful bank thus contains both high-quality ejaculates for immediate AI and strategically important samples which may require ICSI or future technologies [32].

**Table 1:** Sperm-based interventions, demonstrated outcomes, and evidence readiness for endangered-species conservation.

Intervention	Species/Application	Demonstrated Outcome	Evidence Readiness	Principal Limitation	References
Fresh semen and AI	Critically endangered kākāpō	AI-confirmed offspring; four chicks identified through paternity testing	Cat. 2: Live-offspring production in the target or closely related species	Fertility varies; effectiveness depends on reproductive timing and semen quality	[6]
Sperm cryopreservation	Eastern tiger salamander; dusky gopher frog; Puerto Rican crested toad	All three species: F1 offspring produced using cryopreserved sperm; selected F1 adults subsequently produced F2 offspring	Cat. 2: Live-offspring production in the target or closely related species	F2 reproductive competence and population-level benefits require further evaluation	[42]
Post-mortem epididymal sperm recovery	Iberian ibex and mouflon	Epididymal sperm recovered and evaluated after cryopreservation	Cat. 3: Gamete- or embryo-level proof of concept	Post-mortem interval, sperm quality, and lack of demonstrated offspring from recovered epididymal samples in this study	[43]
ICSI using stored sperm	Xenopus laevis experimental model	Viable, reproductively competent offspring following ICSI using long-term cryopreserved sperm	Cat. 5: Indirect evidence from domestic or laboratory models	Species-specific oocyte activation, culture, and reproductive validation	[44]
Species-adapted semen cryopreservation	Banteng and lowland anoa	Species-specific semen characterization and post-thaw sperm survival evaluated, including extender comparisons in banteng	Cat. 3: Gamete- or embryo-level proof of concept	Fertilizing competence and offspring production following cryopreservation requires further validation	[45]
Genomically prioritized semen banking	Proposed integration of semen banks with conservation breeding	Strategic donor representation and future genetic use proposed; no corresponding reproductive outcome tested	Cat. 6: Theoretical or proposed application	Requires genomic prioritization, validated storage, reproductive use, and monitoring	[45]

Abbreviations: AI, artificial insemination; Cat., category; IVF, in vitro fertilization; ICSI, intracytoplasmic sperm injection; F1, first filial generation; F2, second filial generation. Evidence-readiness categories follow the definitions in Section 3 and describe the evidence for the specified species and application, rather than the universal readiness of a technology. Live-offspring production does not independently establish routine conservation use, offspring fertility, or sustained genetic contribution.

The evidence-readiness classification in Table 1 distinguishes between demonstrated reproductive outcomes and preliminary technical feasibility and evidence from experimental models. Artificial insemination produced offspring with confirmed AI-derived paternity in the critically endangered kākāpō, supporting Category 2 evidence [6]. In eastern tiger salamanders, dusky gopher frogs, and Puerto Rican crested toads, cryopreserved sperm produced F1 offspring, and selected F1 adults subsequently produced F2 offspring, supporting Category 2 classification for each species [42]. Conversely, studies that show the recovery and preservation of epididymal sperm in wild ruminants provide evidence at the gamete level, but do not demonstrate the production of offspring using that specific procedure [43]. Although ICSI with long-term frozen sperm produced viable offspring in Xenopus laevis, these results are only indirect evidence for applications to endangered species, as similar outcomes have not been demonstrated in the species for which conservation is intended [44]. Similarly, sperm-quality and cryopreservation studies in banteng and lowland anoa provide species-specific evidence but do not independently validate the proposed genomic prioritization of semen banks [45]. Therefore, evidence readiness should be based on the species being investigated, the proven functional outcome and their relevance to the conservation use-case, rather than solely on technological complexity.

## 5. Oocyte Recovery, IVF, ICSI and Embryo Transfer

Female gametes and embryos are genetically more complete than semen, but they are more technically challenging. Oocytes can be collected from excised ovaries, laparoscopically or transrectally/transvaginally by ovum pick-up. For rare species, the latter approach needs to be engineered around anatomy [46] and anesthesia rather than being mimicked from livestock. Oocytes at different stages may be present in follicles, and IVM competence is affected by follicular size, season [24], donor age, transport time and medium composition. Evaluation of cytoplasmic maturation is especially difficult, with the risk of seemingly mature oocytes fertilizing poorly or arresting early.

Molecular and cytoplasmic features are essential for oocyte developmental competence that might not be completely reflected by conventional morphological assessment. Vitrification in a domestic bovine model was associated with increased reactive oxygen species and abnormalities in meiotic spindle organization. Glutathione ethyl ester supplementation during in vitro maturation decreased cytoplasmic and mitochondrial oxidative stress, helped preserve mitochondrial distribution, and improved developmental competence past the eight-cell stage [47]. However, the study did not reveal statistically significant improvement in Day-8 blastocyst formation compared to vitrified controls. These findings provide experimental evidence of the importance of oxidative balance and mitochondrial organization in bovine oocyte cryobiology, but do not establish equivalent protective effects, or validated biomarkers of competence, in endangered wildlife. Thus, oxidative status, mitochondrial distribution, and spindle integrity should be considered as candidate assessment endpoints, the predictive value of which needs to be independently validated against subsequent developmental and reproductive outcomes in target species.

Molecular evaluation might improve embryo selection, but invasive measures tend to be impractical when embryos of endangered species are limited. A label-free quantitative phase-imaging system, together with machine learning, identified nuclear features associated with embryo-health classifications [48]. These non-invasive approaches could complement traditional morphological grading but need to be validated in each target species before conservation use.

In conventional IVF, successful fertilization requires capacitation of the sperm, zona pellucida interaction, sperm–oocyte fusion and oocyte activation. ICSI bypasses many of the sperm-related hurdles and is emerging as a key component of rhinoceros reproductive rescue programs, particularly when limited quantities of cryopreserved sperm from genetically valuable males must be used efficiently. Sperm injection, however, does not ensure successful oocyte activation and subsequent embryonic development, and species-specific activation and culture conditions may be needed [12]. Embryo culture must also accommodate developing metabolic requirements and gene-expression regulation. Transcriptomic analysis in domestic cattle showed that embryonic transcription was detected from the four-cell stage and the eight-cell stage had the highest proportion of gene activation [49]. These results provide mechanistic insight into bovine embryonic genome activation but should not be interpreted as indicative of similar developmental timing in endangered wildlife. Therefore, maternal transcript clearance, embryonic transcription, and stage-specific gene-expression patterns can be informative for comparative studies of embryo competence, but their timing and predictive significance must be validated independently in target wildlife species.

Embryo transfer (ET) is often a limiting step in the process, because its success is dependent on recipient availability, uterine health, luteal support, synchrony between the embryo and uterus, maternal-fetal interactions and placentation. Interspecific or intersubspecific surrogacy might be possible if body size, gestation length, placental type and endocrine signals are compatible, but this adds another layer of uncertainty. In cheetahs, transfer of in vitro fertilized (IVF) embryos into synchronized recipients resulted in the birth of two cubs, providing direct evidence that IVF and ET can produce live offspring in this species [50]. In the white rhinoceros rescue program, in vitro-produced embryos were transferred and established a pregnancy in a southern white rhinoceros [51]. The original institutional report stated that two southern white rhinoceros embryos were transferred into a southern white rhinoceros surrogate, and the pregnancy was confirmed post-mortem after the death of the surrogate due to a systemic clostridial infection [52]. These examples show that success in pregnancy establishment and live offspring production are different endpoints and that success in one species does not guarantee success in another.

Oocyte and embryo systems should be assessed with stage-specific denominators. Reporting only blastocyst rate may mask low recovery, maturation or fertilization efficiency, whereas reporting only pregnancy rate may mask the number of embryos and recipients required. Conservation studies must encompass the entire development funnel, including follicle aspiration, oocyte retrieval, oocyte maturation, zygote activation, embryo cleavage, blastocyst formation, embryo transfer, pregnancy establishment [53] and birth of offspring. Such transparency is important for protocol comparison and for estimation of the number of donor procedures needed for a population target.

The importance of reporting stage-specific denominators is exemplified by the reproductive programs for the white rhinoceros and the Baudet du Poitou donkey. In white rhinoceroses, 65 ovum pick-up procedures in 22 females resulted in the recovery of 402 oocytes from 1171 aspirated follicles (34.3%). Of 393 oocytes cultured, 150 reached metaphase II (38.2%), 75 cleaved (19.1%), and 51 developed into blastocysts (13.0%). These results show that successful embryo production occurred despite substantial developmental losses [12]. These numbers represent pooled results from southern white rhinoceros, northern white rhinoceros and hybrid embryo production groups and not the full developmental history of embryos used in the subsequent proof-of-concept pregnancy. That pregnancy followed transfer of two in vitro-produced southern white rhinoceros embryos into a surrogate but did not result in a live birth, as documented in the original institutional report. Oocyte-recovery, maturation, and fertilization denominators specific to those two embryos were not provided in that report [52]. Consequently, the pooled 13.0% blastocyst rate from the broader white rhinoceros embryo-production program cannot be attributed to that pregnancy or interpreted as the efficiency of its specific embryo-production pathway [12]. In Baudet du Poitou donkeys, intracytoplasmic sperm injection yielded eight blastocysts from 19 injected oocytes (42.1%), and transfer of three blastocysts resulted in one transient pregnancy detected on embryonic Day 12 but no longer evident on Day 14 [53]. Although delayed meiotic maturation was reported, overall maturation and separate fertilization rates could not be established from the publicly accessible study information. Therefore, the blastocyst rate per injected oocyte should not be interpreted as either a maturation or fertilization rate. The results of this investigation are comparative equid evidence rather than direct validation in endangered wildlife, as this investigation was conducted on an endangered domestic breed. In sum, these examples show that embryo production and pregnancy establishment should be considered in the light of available developmental denominators, the species under study, and the outcomes of pregnancies and offspring, before making conclusions about reproductive efficiency or conservation applicability.

Recipient management is not a veterinary service but a central research domain. Endocrine profiles, ultrasonographic features and, when possible, endometrial molecular markers [54] can be used to assess uterine receptivity. Synchrony windows can be smaller than what domestic relatives may expect. In long-gestation species, support of recipient health must span months or years, with contingency plans for obstetric intervention and neonatal care. Because chronic stress can alter endocrine function [55] and postnatal behavior, temperament, social compatibility and reproductive status should be criteria for selecting surrogates. The ultimate test is a healthy, fertile offspring that can express species-typical behaviors and reproduce in the managed or wild population.

## 6. Cryobiology and Biodiversity Biobanking

Biobanks make biological time a resource for management. Properly curated collections can link founders with descendants decades later, enable retrospective genomic analysis, provide material for disease studies and create options that do not yet exist technologically [5]. The most valuable repositories are not stand-alone frozen collections but linked systems containing provenance, pedigree, phenotype, health status, collection conditions, genomic data [56], legal permissions and intended uses. Without such metadata, samples may be scientifically uninterpretable or legally unusable.

The strategy for cryopreservation should be appropriate for biological material [57]. For example, sperm and dissociated cells tend to be more resistant to controlled-rate freezing than large, lipid-rich oocytes. Embryos are slow-frozen or vitrified depending on the developmental stage and species. Ovarian and testicular tissue can have immature germ cells and somatic niches and provide a route in the future for transplantation or in vitro culture. Viable fibroblasts are important because they support SCNT [58] and iPSC derivation [59]. Establishment of primary cell cultures from skin and testicular tissues of fishing cats further demonstrates the practical value of somatic-cell biobanking for threatened felids [60]. Cryopreservation protocols must be adapted to the distinct thermal and osmotic sensitivities of reproductive cells and tissues across vertebrate species.

The assessment of cryopreservation injury should be based on functional and, where appropriate, molecular endpoints rather than morphological survival alone. Experimental results in domestic ram sperm suggest that oxidative stress, mitochondrial dysfunction, lipid peroxidation, apoptosis-like changes, and DNA fragmentation are important indicators of damage to sperm after thawing [35]. In addition, studies on bovine oocytes have reported changes in oxidative status, meiotic spindle organization, mitochondrial distribution and some gene expression patterns related to vitrification [47]. These findings provide mechanistic evidence that could guide the selection of candidate quality-control parameters for preservation of reproductive material. However, the molecular responses observed in domestic species are not directly transposable to endangered wildlife and these parameters should not be considered as universally validated predictors of reproductive competence. Therefore, quality-control procedures for cryopreservation should be species-specific and tailored to the biological material, and molecular indicators should be interpreted according to functional outcomes such as sperm fertilizing capacity, embryo development, pregnancy establishment and offspring production where possible.

Quality assurance should include identity testing, sterility, viability, post-thaw functional assays [61], duplicate storage sites and contingency plans for liquid nitrogen failure. Genomic optimization is just as crucial. Table 2 gives proposed minimum metadata and sampling criteria. A collection of related individuals can generate an illusion of security, but it preserves little effective diversity [62]. Conversely, a few spare but strategically chosen samples from unique or locally adapted lineages may be of exceptional future value.

The biobanking strategy should separate archival security from reproductive usability. DNA extracts preserve sequence information but cannot currently support organism production; fixed tissues support histology but not cell culture; viable fibroblasts enable reprogramming [59] but do not independently preserve the full range of mitochondrial diversity unless cytoplasmic donors are also represented. Gametes and embryos have the most immediate reproductive value but are often hard to obtain. A complementary biobanking strategy is therefore preferable where each encounter is used to gather multiple types of material under a harmonized consent and permit framework [63]. This redundancy is a hedge against technological uncertainty and enables future techniques to use samples collected today [5].

In the proposed decision framework, biobanking priorities should be based on genetic value, availability of reproductive material, technological readiness, feasibility of collection, animal welfare and likelihood of future reproductive use. Where validated artificial insemination or fertilization protocols are available, semen banking should be a priority for genetically underrepresented males, as it provides a relatively direct route to future reproductive use [45]. Where appropriate donors, established laboratory procedures and compatible recipients exist, oocytes and embryos should be given priority, especially when the preservation of maternal genetic contributions is important [12]. Viable somatic cells provide an alternative means of preserving valuable nuclear genomes for potential future applications, including somatic cell nuclear transfer and induced pluripotent stem cell technologies [5], when usable gametes cannot be obtained. However, preservation of somatic cells should not be confused with the banking of functional gametes, as downstream reproductive applications require further experimental procedures and species-specific validation. Collection of complementary biological materials from the same individual may reduce the risk associated with reliance on a single preservation strategy where possible [63]. Final prioritization should include founder representation, relatedness, anticipated genetic contribution, storage quality and downstream reproductive capacity and collection decisions should be reassessed periodically as conservation needs and technological capabilities change.

Long-term governance needs regular viability audits and transparent prioritization. Stored biological materials should undergo periodic quality assessment rather than remain in indefinite storage without monitoring. Representative vials can be thawed to test viability, and inventory data reconciled with the living population to identify missing founders or geographic gaps. Distributed repositories reduce physical risk, but interoperability is required to ensure that metadata, nomenclature and access rules are consistent [64]. The international networks should also invest in regional capacity, because shipping all samples to wealthy institutions can create inequitable control and delays during conservation emergencies.

**Table 2:** Minimum design and metadata requirements for conservation biobanks.

Domain	Minimum Requirement	Rationale	References
Identity	Unique specimen and individual identifiers; chain of custody	Prevents sample misassignment	[65]
Biological provenance	Taxon, sex, age, pedigree, location, phenotype	Enables interpretation and genetic prioritization	[66]
Health and biosecurity	Clinical status, pathogens, treatments	Supports safe transfer and release	[67]
Collection protocol	Time, anesthesia, tissue, medium, temperature, post-mortem interval	Explains variability and reproducibility	[68]
Cryopreservation	Cryoprotectant, cooling/warming curves, container and storage site	Determines survival and future usability	[69]
Quality control	Pre-freeze and post-thaw viability/function; sterility	Distinguishes stored quantity from usable material	[61]
Genomics	Genotype, kinship, ancestry, load and adaptive markers	Optimizes representation and mating	[70]
Governance	Ownership, permits, access, benefit sharing and use restrictions	Ensures legal and ethical usability	[71]
Resilience	Duplicate storage, alarms, emergency supply and audit schedule	Reduces catastrophic loss	[72]

## 7. Cloning, Stem Cells and In Vitro Gametogenesis

Cell-based technologies provide possible alternatives when founders die before reproductive techniques become available or when gametes are not available; however, most are experimental, and SCNT has only been successfully applied in a small number of conservation programs. In SCNT, a somatic nucleus is transferred into an enucleated oocyte to generate an embryo that carries the nuclear genome of the somatic-cell donor, while its mitochondrial genome is primarily from the recipient oocyte. A bioRxiv preprint reported the production of cloned black-footed ferrets from cryopreserved cells of a deceased female with no descendants in the living population; this evidence should be interpreted cautiously because the cited version had not undergone peer review [73]. Similarly, cloning of Przewalski’s horses from cryopreserved somatic cells for decades has shown that it is possible to recover underrepresented nuclear genetic variation [5]. These examples demonstrate the potential of SCNT to recover valuable founder genomes, although offspring production alone does not demonstrate sustained genetic contribution or population-level conservation benefit.

SCNT is inefficient and is accompanied by abnormal reprogramming, pregnancy loss, placental pathology [9] and neonatal morbidity [74]. It should therefore be limited to genotypes with high conservation value and undertaken under explicit welfare thresholds. Cloning alone cannot recreate a population, as it requires multiple unrelated genomes, sex balance, reproductive competence, and ecological conditions. Its best near-term application is to reintroduce lost founder genomes into an extant managed population followed by natural or assisted breeding.

A major limitation of SCNT is incomplete molecular reprogramming. A multi-omics comparison of primate SCNT and ICSI embryos showed widespread DNA hypomethylation and loss of imprinting in maternally imprinted genes in SCNT blastocysts. The same study identified abnormal extraembryonic development as a contributor to post-implantation failure and demonstrated that trophoblast replacement could improve developmental outcomes following SCNT [9]. Interspecies experiments have also shown that incompatible mitochondrial DNA and lack of cytoplasmic reprogramming factors can limit nuclear replication, pluripotency-gene expression and blastocyst development [10]. Thus, molecular evaluation of SCNT embryos should include assessment of epigenetic status, genomic stability, mitochondrial function, embryonic genome activation and placental-lineage development.

The use of iPSCs and IVG for the recovery of reproductive potential from archived somatic cells is a promising experimental approach but is in its infancy for application in endangered wildlife. In the northern white rhinoceros, somatic cells have been reprogrammed into pluripotent stem cells, suggesting the possibility of generating cellular resources for future reproductive studies [4]. Further studies derived primordial germ-cell-like cells from rhinoceros pluripotent stem cells, showing species-relevant direct evidence of early germ-cell differentiation [7]. However, these achievements do not demonstrate completion of meiosis, establishment of normal genomic imprinting, production of functional gametes, or generation of offspring. By contrast, experimental IVG systems with mouse pluripotent stem cells have been able to generate functional oocytes, demonstrating reproductive feasibility in laboratory mice [75]. But this is circumstantial evidence from model species and cannot be considered evidence of technological readiness in endangered wildlife. Species-specific differences in germ-cell specification, meiotic progression, epigenetic reprogramming and gonadal support remain major translational barriers. As such, wildlife applications of iPSC-derived germ cells and IVG should remain experimental until their functionality and reproductive outcomes can be validated in the species of interest. Genome editing may provide additional experimental options for studying or even correcting well-characterized deleterious variants, but its use in conservation should be distinguished from genetic rescue through the restoration or redistribution of existing variation and from speculative applications involving enhancement or de-extinction.

Conservation genetics of cloning is different from agricultural replication. In production systems, cloning often increases an elite genotype. In conservation, the primary objective is instead to recover missing or underrepresented genotypes and facilitate their contribution to the existing population. Prior to SCNT, donor identity, ancestry, sex and predicted genetic contribution should be confirmed by whole-genome data [76]. Mitochondrial-nuclear compatibility is also a factor, since the clone inherits its mitochondria from the oocyte donor [10]. Epigenetic and health assessments should extend into adulthood, while reproductively competent, cloned individuals should be incorporated into appropriate breeding programs to facilitate the transmission of their genetic variation to subsequent generations.

Stem-cell routes require rigorous benchmarks ahead of use of endangered animals. Pluripotency markers alone are insufficient evidence for germline competence. Candidate cells must remain chromosomally stable, undergo proper epigenetic resetting and meiosis, establish sex-specific genomic imprints, and produce functional gametes capable of supporting normal fertilization and development [77]. Reproductive organoids and xenograft gonadal niches are among the experimental model systems used to study germ cell differentiation today [78], but their conservation application remains theoretical. A pragmatic near-term strategy is to generate genetically diverse and well-characterized iPSC panels from endangered species. In northern white rhinoceroses, induced pluripotent stem cell-derived primordial germ-cell-like cells expressed molecular markers associated with early germ-cell differentiation [7]. However, the expression of these markers does not demonstrate meiotic competence, appropriate genomic imprint establishment, functional gamete production, or the capacity to support normal embryonic development and offspring production. Thus, conservation programs should preserve diverse cell lineages and not claim that iPSC-derived gametes are ready for immediate species restoration. Their reproductive application in endangered wildlife will require validation of functional gamete production, developmental competence, offspring health and fertility, and acceptable reproductive safety.

## 8. Integrating ART with Conservation Genomics and Genetic Rescue

Genetic rescue is the intentional introduction or restoration of genetic variation to increase population fitness and long-term viability [11]. It is not equivalent to genetic restoration, which is the reintroduction of historical or underrepresented genetic variation without necessarily demonstrating an immediate fitness benefit [79]. Assisted gene flow involves the intentional movement of individuals or reproductive materials between populations to facilitate genetic exchange. Genomics-informed donor selection uses genome-wide information to identify genetically and ecologically appropriate donors [18]. Founder-genome recovery is the restoration of nuclear genetic variation of an underrepresented or deceased founder by using banked gametes, embryos or viable somatic cells [5]. Furthermore, these strategies are distinct from de-extinction, which seeks to create an organism similar to an extinct taxon rather than support an extant population [80]. Whole genome data can inform these interventions by providing estimates of kinship, runs of homozygosity, deleterious genetic load, ancestry and population structure [81]. But neutral diversity should not be confused with adaptive variation. Increasing genome-wide heterozygosity can reduce inbreeding, but retention of locally adaptive variation may be necessary for survival in specific ecological contexts. Sex-linked and mitochondrial diversity should also be considered, as reproductive technologies do not equally transmit all genomic components; for example, semen-mediated gene flow transfers paternal nuclear variation but not the donor’s mitochondrial genome [38].

There is a need for conceptual clarity; here, genomic rescue is used as an operational term for genomics-informed conservation strategies that aim to preserve, recover or redistribute genetic variation via donor selection, assisted gene flow, reproductive technologies and the use of banked biological materials. Genomic rescue describes a genomics-informed conservation strategy and does not, by itself, establish a successful conservation outcome. In contrast, genetic rescue requires evidence of improved population fitness or viability following the introduction of genetic variation [11]. Genomic allocation in this context is the prioritization of donors, preserved biological materials and breeding combinations based on relatedness, founder representation and genetic diversity [18]. Unlike these approaches, genome editing involves targeted modification of DNA sequences, rather than transfer or recovery of existing genetic variation. Although editing might have applications for future research, the technical development, experimental validation and implementation in conservation are outside the scope of this review, which focuses on vertebrates. Genome editing is thus not considered an intervention in the proposed genetic-rescue pathway, which instead focuses on genomics-informed donor selection, assisted gene flow, founder-genome recovery, and subsequent assessment of genetic and population-level outcomes.

Whole-genome sequencing, demographic reconstruction and climatic-niche modeling were combined to compare potential donor populations in montane red foxes based on genomic diversity, inbreeding, demographic history and environmental fit [18]. This implies that donor selection should combine runs of homozygosity, deleterious genetic load, ancestry, and adaptive variation with reproductive feasibility, disease risk, and ecological compatibility, instead of geographic proximity or overall genetic distance only.

**Figure 3:**
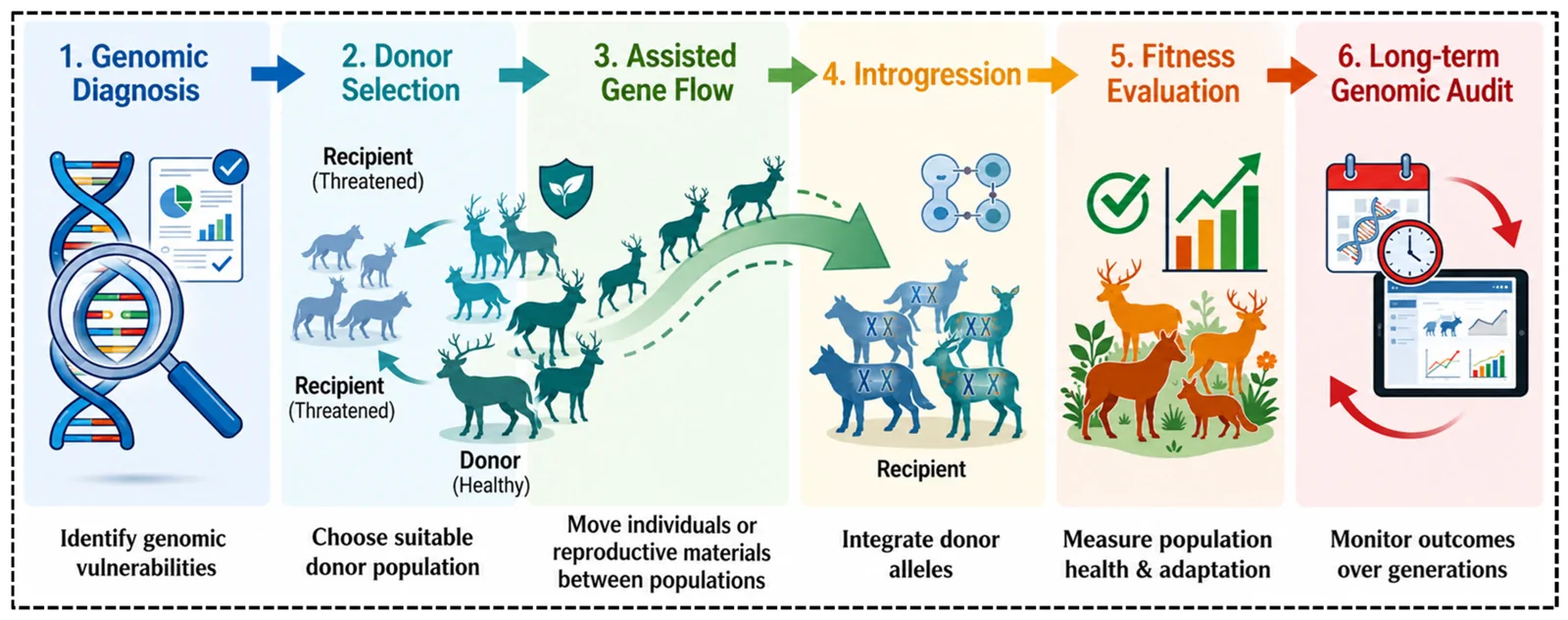
Genomics-informed framework for assisted gene flow and genetic rescue in endangered vertebrates. The framework includes six interlinked stages: (1) genomic diagnosis, (2) donor selection, (3) assisted gene flow, (4) introgression, (5) fitness evaluation, and (6) long-term genomic audit. The genomic assessment supports identifying suitable donor populations by genetic diversity, relatedness, ancestry and adaptive compatibility. Assisted gene flow is the movement of individuals or reproductive material among populations, and introgression is the incorporation of donor alleles in subsequent generations. Fitness assessment and long-term genomic monitoring evaluate reproductive success, offspring fitness, genetic contribution and population-level outcomes. The framework aims to reshuffle existing genetic variation and does not involve genome editing or CRISPR-mediated modification. The pathways are conceptual and do not imply that gene flow necessarily leads to successful genetic rescue. Top arrows indicate stage progression. Teal arrows indicate donor-to-recipient transfer, green arrows indicate gene flow and introgression, and red circular arrows indicate repeated genomic monitoring.

The best-known empirical case of genetic rescue is the Florida panther. Recent genomic analysis has clarified the restoration of variation and the complexity of ancestry-dependent fitness [82]. Other comparable work on mountain lions [83], Scandinavian wolves [84], adders [85], prairie chickens [86] and island populations [87] indicates that gene flow might increase survival or reproductive success but might also decrease local adaptation or provide a short-term advantage if the population remains small. Rescue is therefore a process, not a single translocation. Figure 3 illustrates how genomic information can contribute to donor selection, assisted gene flow, introgression and long-term evaluation of genetic-rescue results.

Assisted reproductive technologies can facilitate genetic exchange without relocating whole animals, as demonstrated by artificial insemination using genetically underrepresented males in the endangered kākāpō [6]. Semen can reduce disease and behavioral risks, embryos can transmit both parental genomes, and cloning can revive alleles from donors no longer present. But the likelihood that a rescued genome will contribute to future generations needs to be modeled. In addition to census numbers, founder equivalents, mean kinship, effective population size and allele retention should be monitored [88]. Table 3 summarizes the principal rescue modalities, benefits and failure modes.

Donor selection should consider continued inbreeding, risk of outbreeding, disruption of local adaptation, loss of population identity, and introduction or expression of deleterious variants. Whole-genome simulations allow for comparison of candidate donors under alternative mating and population-growth scenarios [89]. Donors should be selected based on ancestry, relatedness, runs of homozygosity, deleterious genetic load, and locally adaptive variation, not geographic proximity or overall genetic distance alone [81]. The number of donors should also be optimized, as multiple suitable donors are generally less susceptible to reproductive failure and genetic drift than contributions of a single donor [90]. But a single archived founder may still be useful if it contains otherwise missing or underrepresented variation.

Post-rescue management ascertains if the genetic and demographic advantages are ephemeral or enduring. An elevation in heterozygosity by itself does not indicate effective genetic rescue; the progeny of introduced or restored founders must endure, reproduce, and pass on their lineage across several generations while enhancing population viability [11]. Genomic surveillance should thus evaluate the continuity of ancestry, actual relatedness, allele preservation, and variations in harmful homozygosity across consecutive generations [88]. These strategies must be assessed in conjunction with reproductive success, recruitment, population expansion, and ongoing ecological challenges, as genetic improvements cannot perpetually offset mortality resulting from disease, habitat deterioration, or other external stressors [91].

The conservation relevance of reproductive and genetic-rescue technologies depends on their readiness for species-specific application as well as the biological outcomes they demonstrate. Different levels of validation of technology, such as successful embryo development, production of offspring, or genetic rescue, should not be seen as equivalent measures of conservation success. For example, somatic cell nuclear transfer has enabled recovery of historically underrepresented nuclear genomes and generation of live offspring, although further evaluation is needed to determine subsequent reproductive competence and sustain genetic contributions to managed populations [5]. Similarly, embryo production and transient pregnancy after embryo transfer indicate technical feasibility but do not demonstrate reliable live-offspring production or long-term reproductive success [53]. Technological readiness should therefore be considered along with reproductive efficiency, animal welfare, recipient compatibility and the possibility of continuing genetic contribution. These considerations make the distinction between experimentally demonstrated outcomes and the additional validation and implementation requirements needed to translate reproductive technologies into measurable conservation benefits.

## 9. Taxon-Specific Translation Across Vertebrates

The key biological challenge is taxonomic diversity. Mammalian ART has a wealth of domestic-animal knowledge, but reproductive anatomy and endocrine physiology still differ significantly among carnivores, ungulates, elephants, marsupials and bats. Felids exhibit variable sperm pleiomorphism [92] and ovulatory mechanisms [93]; canids may exhibit mono-estrus and prolonged oocyte maturation [94]; rhinoceroses require specialized ovum pick-up and ICSI; and elephants experience major constraints in cycle length, uterine access and gestation [95]. Thus, success relies on comparative physiology and species-specific experimentation, not a one-size-fits-all wildlife protocol.

The evidence for reproductive technologies varies widely among taxonomic groups and significantly among individual species within each group. Species-specific semen preservation and artificial insemination have been used in selected conservation programs in mammals, whereas oocyte and embryo technologies are often at developmental proof-of-concept stages. Artificial insemination has been used to produce offspring in threatened species of birds, while more advanced primordial germ-cell applications have achieved different functional endpoints in domestic and nondomestic avian models. Fish germ-cell transplantation has demonstrated the production of gametes from donor cells and, in some experimental systems, the production of offspring, but transfer to endangered species still requires compatible recipients. The cryopreservation of amphibian sperm has played a key role in the production of offspring in some selected species, while reproductive applications in reptiles remain largely in earlier experimental stages. These differences emphasize the importance of assessing technological readiness in relation to species-specific reproductive endpoints, rather than broad taxonomic or technical classifications.

Semen cryopreservation, AI and PGC technologies are complementary approaches for avian genetic-resource preservation. The avian embryo develops in a shelled egg, so the standard mammalian embryo-transfer procedures cannot be applied directly. Alternatively, cultured PGCs can be transplanted into recipient embryos to create germline chimeras capable of producing donor-derived gametes. The experimental chicken system showed that cryopreserved PGCs are fertile, which was successfully applied to produce donor-derived offspring for germline chimera production and subsequent breeding in indigenous Chinese chickens [13]. Furthermore, the transplantation of chicken PGCs into sterile interspecific avian recipients resulted in germ-cell colonization and production of mature sperm, providing experimental proof that recipient species can support donor-derived gametogenesis [96]. But these are results from experimental systems based on chickens and do not demonstrate routine germline restoration in endangered wild birds. The translation to threatened avian species still requires successful PGC isolation and culture, recipient compatibility, germline transmission and production of reproductively competent offspring. As a consequence, chicken evidence must be separated from direct conservation outcomes, and PGC-based technologies should be classified based on functional endpoints shown in individual target species.

Fish provide powerful systems for germ-cell transplantation. Spermatogonia or germ cell primordia from endangered donors can engraft in sterilized recipients and thereafter generate donor-derived gametes. This way, generation time and body size constraints can be minimized if a small, fast-maturing surrogate is available. Functional gametes have been generated from cryopreserved testicular cells after transplantation in a number of models [97]. Amphibian programs emphasize hormone-induced spermiation, sperm freezing and IVF, with increasing focus on post-thaw fertilization rather than motility alone [15].

Reproductive technologies are biologically feasible with different developmental outcomes and conservation applicability for different vertebrate species. Their readiness to provide evidence should be judged on the basis of the results obtained for specific interventions, rather than by generalizing across taxonomic groups. The evidence available (summarized in Table 4) includes: routine use in conservation applications, production of live offspring, proof of concept at the gamete or embryo level, proof of concept at the cellular or molecular scale, indirect evidence from domestic or laboratory models, and theoretical or future applications. These distinctions are important because the ability to successfully preserve gametes, develop embryos or produce offspring does not necessarily translate to routine applicability or long-term conservation benefit [6,12]. Further validation should include species-specific reproductive constraints, technological feasibility, health and fertility of offspring, and potential for continued genetic contributions to managed populations.

Reproductive technologies in reptiles, although important for conservation, still remain underdeveloped. Reproductive monitoring and intervention are confounded by sperm storage in the female reproductive tract, temperature-dependent sex determination [98] and poorly characterized endocrine cycles. Semen collection and short-term storage are improving in snakes [99], crocodilians and chelonians, but reliable offspring production with cryopreserved sperm is still rare.

Cross-taxon learning still has value, if it is mechanistic, not protocol-based. Even if concentrations and timing differ, principles of membrane phase transitions, osmotic injury, oxidative stress [35,47], meiotic competence and species-specific reproductive physiology can inform comparative experiments. Phylogenetic patterns could be identified by standardized repositories of negative and positive results. Conservation centers also should develop long-term partnerships with livestock, aquaculture, zoo, botanical and human reproductive laboratories, while maintaining wildlife-specific welfare and population goals.

**Table 4:** Evidence readiness, reproductive bottlenecks, and validation priorities across major vertebrate groups.

Taxon	Demonstrated Outcome (Readiness)	Major Bottleneck	Next Validation Requirement	References
Mammals	Markhor: embryo-level proof of concept (Cat. 3)	Oocyte competence and recipient compatibility	Pregnancy and live-offspring production	[100]
Birds	Kākāpō: AI offspring (Cat. 2); chicken: PGC offspring (Cat. 5); rock dove: PGC culture (Cat. 4)	Germline access and species compatibility	Functional germline transmission in threatened birds	[6,13,101]
Fishes	Bluefin tuna: donor-derived sperm (Cat. 3); nibe croaker: donor-derived offspring (Cat. 5 for endangered-species application)	Recipient sterilization and donor-cell colonization	Offspring production in target species	[102,103]
Amphibians	Salamander: offspring from cryopreserved sperm (Cat. 2)	Cryotolerance and larval survival	Adult fertility and reproductive contribution	[104]
Reptiles	Beauty snake: post-thaw sperm viability (Cat. 3)	Reproductive cycles and cryobiology	Fertilization and live offspring	[99]

Abbreviations: AI, artificial insemination; Cat., category; PGC, primordial germ cells. Evidence-readiness categories follow Section 3: Cat. 1, routine use in conservation; Cat. 2, live-offspring production in the target or closely related species; Cat. 3, gamete- or embryo-level proof of concept; Cat. 4, cellular or molecular proof of concept; Cat. 5, indirect evidence from domestic or laboratory models; and Cat. 6, theoretical or proposed application. Categories apply to the species and intervention specified within each row, rather than to the taxonomic group as a whole.

## 10. Welfare, Ethics, Governance and Resource Allocation

The ethical justification for reproductive interventions depends on whether the anticipated conservation benefit outweighs the animal welfare risks, invasiveness of the procedure and resource input [105]. To link the evidence compiled in Section 4, Section 5, Section 6 and Section 7 to conservation decisions in practice, each intervention should be chosen according to its species-specific readiness, genetic value, feasibility and downstream requirements. Semen collection, cryopreservation and AI should be prioritized when semen quality, breeding timing and insemination protocols are sufficiently validated, especially when genetic exchange can be achieved without moving animals [100]. In cases where conventional breeding or AI is inadequate and appropriate donors, laboratory protocols, and compatible recipients are available, oocyte retrieval, IVF, ICSI, and ET should be considered [12]. Biobanking is justified where genetically valuable material can be collected and preserved with reliable provenance, quality control and realistic future applications, even if immediate reproductive use is not feasible [63]. SCNT should be restricted to genetically valuable or underrepresented founder genomes for which appropriate oocytes, surrogate capacity and welfare safeguards exist [5]. iPSC approaches and IVG should remain experimental until functional gametes, developmental competence and reproductive safety are demonstrated in relevant species [7]. Of these interventions, priority should be given to approaches with credible prospects for producing healthy, fertile offspring that contribute genetically to the population, rather than to laboratory success alone. Procedures involving anesthesia, repeated gamete collection, hormonal stimulation and surrogate pregnancy require predefined welfare criteria, appropriate oversight and transparent reporting of complications, pregnancy losses and neonatal outcomes [105,106]. Interventions should be delayed when the expected conservation benefit cannot justify animal burden or when critical downstream reproductive and management capacity is not available.

**Figure 4:**
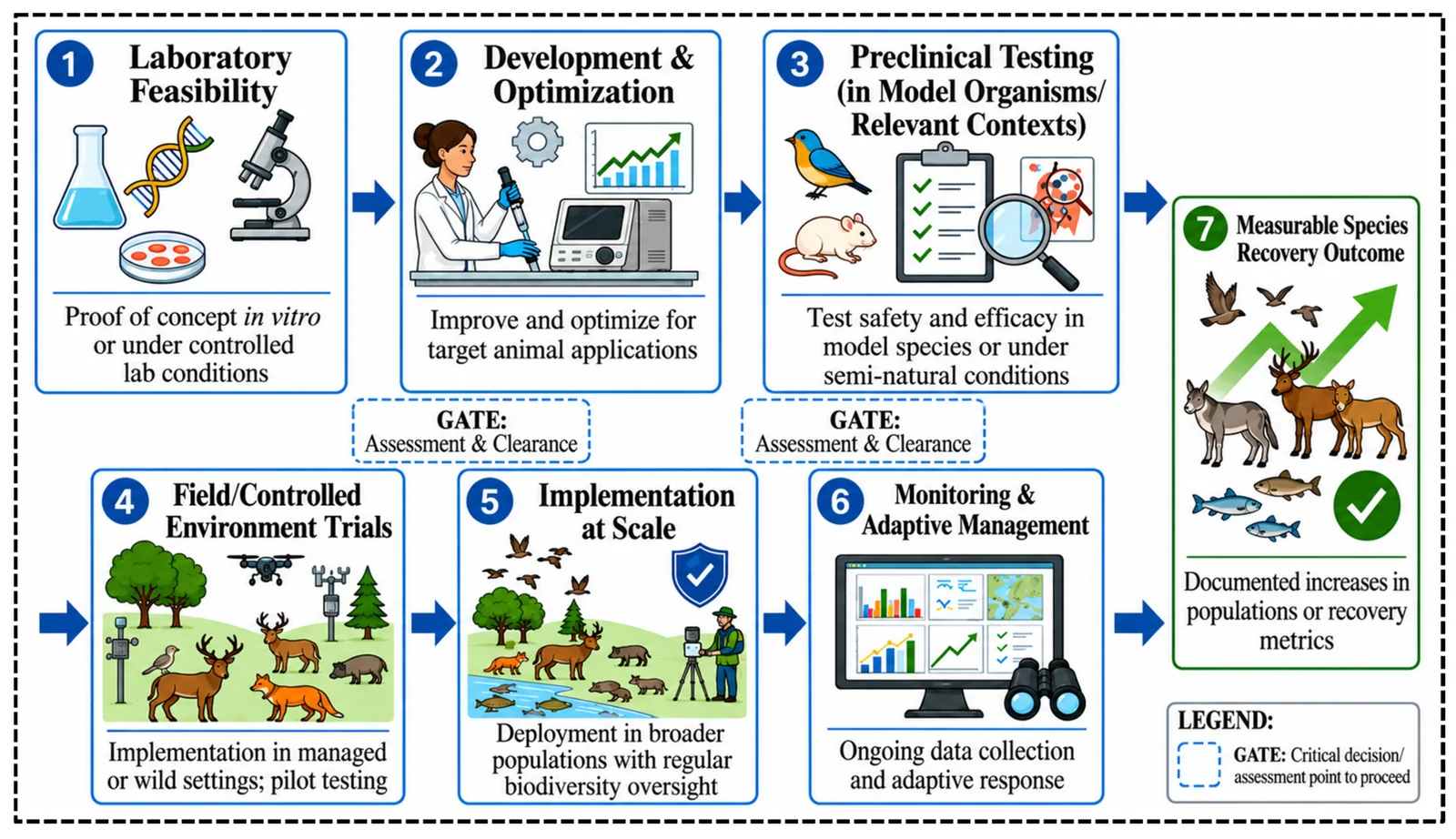
Implementation and validation pathway for translating reproductive technologies into conservation practice. The proposed framework is composed of seven interconnected stages: (1) laboratory feasibility, (2) development and optimization, (3) preclinical testing in relevant models or experimental systems, (4) controlled or field trials, (5) implementation and scaling, (6) monitoring and adaptive management, and (7) assessment of measurable species-recovery outcomes. Arrows indicate the suggested progression between stages, and decision gates represent the technical, reproductive, welfare and conservation requirements that need to be assessed before proceeding to the next stage. These stages refer to the translation of an intervention into conservation practice and should not be confused with the six evidence-readiness categories described in Section 3. Evidence categories describe the demonstrated outcome and its relevance to the species, whereas implementation gates assess whether there is enough validation, safety, reproductive capacity, and conservation justification to advance the intervention. Progression is thus conditional, not automatic and success in laboratory or offspring production alone does not guarantee long-term conservation benefit. Solid blue-bordered boxes indicate implementation stages (1–6), dashed blue-bordered boxes mark decision gates requiring assessment and clearance, and the green-bordered box indicates measurable species-recovery outcomes (7).

Governance matters as well. National wildlife laws, the Convention on Biological Diversity, access-and-benefit-sharing rules, the Convention on International Trade in Endangered Species of Wild Fauna and Flora (CITES), veterinary import controls and institutional agreements may govern the collection, storage and international transfer of wildlife genetic resources. Indigenous peoples and local communities may have rights, knowledge and legitimate interests in species and samples. Biobanks should record ownership, custodianship, permitted uses, benefit sharing and conditions for international transfer [107]. Open science cannot defeat sovereign or community rights [108]. We should not be forced into a false choice between biotechnology and habitat conservation regarding resource allocation. The pertinent question is whether technology provides marginal recovery value within a funded species plan [109]. High-profile rescue projects can generate capacity and public support, but they can also detract from attention to preventable threats. Figure 4 illustrates the progressive stages of readiness required to convert a laboratory procedure into a safe and measurable conservation intervention. A program should only move forward when the next ecological, demographic and welfare requirements are credible [80].

Opportunity cost and distributive justice should be part of ethical assessment [110]. A high-cost intervention might be warranted for a functionally extinct taxon if it creates reusable capacity, but decision makers should reveal what alternative conservation actions are displaced. Cost accounting has to cover not only the laboratory step but also repeated donor procedures, surrogate maintenance, failed cycles, neonatal care, long-term housing and genomic monitoring [111]. Independent review panels can compare predicted genetic gain per cost unit [112] and per animal burden, while noting that cultural and ecological values cannot be converted into a single financial metric.

Public communication should avoid narratives of technological resurrection that imply extinction is reversible. Recovered or engineered animals cannot recreate lost ecosystems, learned behaviors or coevolved communities automatically [113]. Transparent communication should distinguish prevention, genetic reinforcement, functional restoration and de-extinction. This clarity protects public trust and keeps policy focused on preventing habitat loss. It also helps communities evaluate whether a proposed intervention aligns with their values, land-use priorities and relationships with the species [114].

## 11. Research Priorities and Implementation Roadmap

A practical implementation model can be divided into five phases. First, programs should assess the demographic and genetic problem: number and sex of breeders; projected inbreeding; founder representation [115]; reproductive failure; and habitat carrying capacity. Second, a living biobank should be created [116] through regular collection from managed animals, field captures and post-mortem opportunities [117], with duplication across secure facilities. Third, they should choose the least invasive technology that can achieve a specified genetic contribution. Fourth, they should confirm the whole chain by producing healthy and fertile offspring and by being incorporated into breeding plans. Fifth, they should trace descendants in genomic, reproductive, behavioral and ecological dimensions [118].

Priorities for the next decade include non-invasive endocrine sensors [119]; anesthesia-light gamete collection [120]; lipid and membrane profiling to predict cryotolerance [121]; chemically defined media; organoid models of gonadal function [122]; automated embryo imaging [48]; cross-species placental biology; and methods for generating functional gametes from banked cells. Procedures in multi-institutional “model-to-target” pipelines should be solved using related common species prior to exposure of endangered animals. Negative results and protocol details should be published, as hidden failures lead to repeated use of animals. Success metrics need to be standardized. Minimum reporting should include donor identity and genomic value, conditions of sample collection, pre- and post-thaw function, number of oocytes and embryos at each stage, synchronization of recipients, pregnancies [123], losses, health of offspring, fertility and eventual genetic contribution. Population-level outcomes should include change in mean kinship, effective size, inbreeding load, reproductive rate and extinction probability [124]. These metrics enable comparability of technologies and enable adaptive management. Molecular validation should follow these developmental and population-level outcomes. The major molecular barriers, measurable biomarkers, validation endpoints and current evidence supporting the major reproductive technologies considered in this review are summarized in Table 5.

The most fundamental change will be from isolated technical demonstrations to integrated conservation portfolios [125]. The value of a semen bank without insemination capacity, an embryo without a recipient, or a clone without plans for breeding and habitat is limited. In contrast, there are low-tech solutions related to prioritization of genomics and good field management that could have a long-term effect. The field should thus invest in parallel in biological collections, reproductive know-how, genomic analytics, veterinary infrastructure and ecological restoration. Another priority is modeling the population in advance of intervention [126]. Models must estimate the number of successful conceptions, surviving juveniles and reproducing descendants needed to change effective population size or maintain a given allele at a desired frequency [124]. Placing these thresholds within the framework of empirically measured laboratory efficiencies can avoid unrealistic expectations and clarify the value of incremental improvements [6]. The model can then be updated adaptively through management following each collection, fertilization, pregnancy or release event. This quantitative feedback-loop is especially important for long-lived species where a decade of technical activity might otherwise pass before population consequences are apparent [127].

A coordinated research infrastructure will accelerate progress. Shared protocol registries should include common outcome definitions and unsuccessful trials [128,129]. Comparative cryobiology, genomic prioritization, and quality control from reference laboratories could benefit the smaller conservation programs. Training should be embedded in range countries so that sample collection, reproductive monitoring and biobank governance can be sustainable without reliance on visiting teams. Mobile laboratories [130] and validated transport systems [131] would enhance recovery of field mortalities and reduce time between collection and preservation.

Programs should also plan for the obsolescence of technology. Samples stored on proprietary devices or on undocumented media may become unreadable decades later. Open standards for metadata, redundant formats, and retention of raw genomic data can keep the interpretation of data possible [132]. Periodic horizon scanning should determine whether banked tissues are now suitable for new applications [133], but activation should be tied to species-recovery priorities. The value of technological innovation should be assessed primarily by its capacity to produce genetically valuable, healthy, and reproductively competent animals that contribute to secure and viable populations.

**Table 5:** Molecular evaluation of reproductive technologies used in conservation.

Technology	Main Molecular Concern	Required Validation	References
Semen freezing	Oxidative, mitochondrial, and DNA damage	Post-thaw fertility	[35]
Oocyte vitrification	Oxidative and spindle damage	Embryo development	[47]
Embryo culture	Abnormal embryonic genome activation	Stage-appropriate transcription	[49]
SCNT	Epigenetic and imprinting failure	Normal placental and fetal development	[9]
Interspecies SCNT	Nuclear–mitochondrial incompatibility	Embryo and blastocyst development	[10]
iPSC-derived germ cells	Incomplete germ-cell specification	Meiosis and functional gametes	[7]
IVG	Abnormal meiosis or imprint establishment	Fertile and healthy offspring	[75]
Genomic selection	ROH and deleterious genetic load	Genetically suitable donors	[18]

Abbreviations: iPSC, induced pluripotent stem cell; IVG, in vitro gametogenesis; ROH, runs of homozygosity; SCNT, somatic cell nuclear transfer.

## 12. Conclusions

Reproductive biotechnology provides opportunities to conserve, transfer and recover genetic variation that might otherwise be lost from endangered populations of vertebrates. The integrated framework provided in this review connects species-specific reproductive limitations, technological readiness, molecular assessment and genetic management with animal welfare and measurable conservation outcomes. Artificial insemination and semen cryopreservation have been successfully applied in selected species, and IVF, ICSI and embryo transfer need further validation on a species-specific basis. Somatic cell nuclear transfer and stem-cell-based approaches offer additional opportunities to recover underrepresented genetic variation, but their technological readiness and reproductive outcomes are considerably different. Ultimately, the conservation value of these interventions will depend on the choice of genetically appropriate donors, acceptable animal welfare outcomes, the production of healthy and fertile offspring, and sustained genetic contributions to viable populations. Therefore, reproductive biotechnology should be used as a tool to supplement, not supplant, habitat protection, threat mitigation, and long-term population management. Without these foundations, even technically successful reproductive interventions may have limited impact on species recovery.

## Figures and Tables

**Table 3:** Genetic-rescue modalities, evidence readiness, and implementation requirements.

Modality	Application	Main Benefit	Evidence Readiness	Validation/Monitoring Needed	References
Animal translocation	Suitable donors and habitat	Nuclear and mitochondrial gene flow	Cat. 1: Routine use in conservation	Survival, fitness, genetic contribution	[11]
Semen-mediated gene flow	Females accessible; male movement restricted	Paternal genetic exchange	Cat. 2: Live-offspring production in the target or closely related species	Offspring fertility, allele transmission	[6]
Embryo transfer (ET)	Suitable embryos and recipients	Biparental genetic contribution	Cat. 5: Indirect evidence from domestic or laboratory models	Sustained pregnancy, live birth	[53]
SCNT/archived founder recovery	Valuable Somatic cells; no usable gametes	Recovery of founder nuclear genomes	Cat. 2: Live-offspring production in the target or closely related species	Offspring fertility, genetic contribution	[5,9]
iPSC-derived germ cells	Archived somatic-cell resources	Experimental germ-cell derivation	Cat. 4: Cellular or molecular proof of concept	Functional gametes, reproductive safety	[7]
In vitro gametogenesis (IVG)	Future use of reprogrammed cells	Potential functional gamete production	Cat. 6: Theoretical or proposed application	Fertilization, offspring, safety	[7]

Abbreviations: Cat., category; SCNT, somatic cell nuclear transfer; iPSC, induced pluripotent stem cell; IVG, in vitro gametogenesis. Evidence-readiness categories follow the definitions in Section 3 and describe the demonstrated outcomes and their relevance to the specified species and application. Category 2 indicates live-offspring production but does not establish routine conservation use, offspring fertility, or sustained genetic contribution. Category 5 identifies indirect model-species evidence rather than validation in the target wildlife species.

## Data Availability

No new data were created or analyzed in this study. Data sharing is not applicable to this article.
